# An examination of factors that may contribute to gender differences in psychomotor processing speed

**DOI:** 10.1186/s40359-021-00698-0

**Published:** 2021-12-02

**Authors:** Eka Roivainen, Frans Suokas, Anne Saari

**Affiliations:** Department of Medical Rehabilitation, Oulu University Hospital, PL 26, 90029 OYS Oulu, Finland

**Keywords:** Processing speed, Gender differences, Cognition, Motor speed

## Abstract

**Background:**

For unknown reasons, females outperform males on tests of psychomotor processing speed (PS), such as the Coding and Symbol Search subtests of the Wechsler Adult Intelligence Scale.

**Method:**

In the present study, the effects of intelligence, memory, personality, fine motor speed, gross motor dexterity, height, weight, age, sex, and education on psychomotor processing speed were studied in an outpatient sample (*n* = 130).

**Results:**

Moderate (r > .40) correlations were found between PS and verbal reasoning, nonverbal reasoning, verbal memory, and fine motor speed. Weak (r > .20) correlations were found between PS and gross motor dexterity, extraversion, education, weight, and sex. Females outperformed males in PS and in fine motor speed. Stepwise linear regression analysis indicated nonverbal reasoning, fine motor speed, and sex as independent predictors of PS.

**Conclusions:**

One interpretation of the results is that the factors underlying sex differences in processing speed are not psychological but neurological or physiological in nature and therefore a wider variety of measures from these disciplines are needed for further studies. For clinical assessment purposes, psychological tests should preferably provide different norms for male and female PS scores.

## Introduction

Fast performance on simple cognitive tasks such as copying digits and symbols, locating identical pictures, or rapidly naming objects correlates with other cognitive skills such as logical reasoning, vocabulary, and memory [1, 2]. For this reason, psychomotor processing speed (PS) is considered to be one of the factors of intelligence. For example, in the Cattell-Horn-Carroll [3] theory, processing speed is one of the nine major broad abilities, and the predominant intelligence test, the Wechsler Adult Intelligence Scale [4], includes the PS index as one of the four major subscales.

While it is widely agreed that processing speed is an important element of cognition, there is no consensus on the exact nature of this construct. One of the major questions is whether speed is a unitary ability or instead is a collection of several cognitive processes [5, 6]. Based on the observation that performance in reaction time tests is associated with a wide variety of more complex mental skills, Jensen [7] has suggested that mental speed is a basic process that underlies general cognitive ability. However, studies of age-related changes in cognition suggest that a variety of neural systems affect processing speed [8, 9].

Another problematic feature of PS is that there seem to be differences across sex and nationality. For example, on the Wechsler tests (WAIS, WAIS-R, WAIS III and WAIS IV) [1, 10–12], there is a small difference in favor of males on the nonverbal, verbal, and working memory subtests, while females outperform males on the PS tests. In the latest version, WAIS IV, the index scores for males and females were 101.8 and 98.4, respectively, on the Perceptual Reasoning (PRI), Verbal Comprehension (VCI) and Working Memory (WMI) composite indexes and 97.7 and 102.1 on the Processing Speed index (PSI). There are also significant cross-national differences in IQ test profiles. In a recent study [13] the mean PRI scores for Finnish, Scandinavian, German and French WAIS IV standardization samples varied from 104 to 109 while the PSI scores varied from 96 to 101 when scored using U.S. norms (U.S. mean = 100 for all indexes). Thus, the WAIS factor model involving the four index scores is not perfectly consistent across sex and nationality.

Several factors have been suggested to underlie the differences in the PRI/PSI ratio between men and women, and across nations [14]. The size of hands and finger thickness has been shown to affect tasks involving dexterity [15, 16]. In fine motor tasks, persons with narrow digits are at an advantage [17], and females have smaller hands than men do. Recent studies show that populations from cold regions have relatively shorter and wider fingers, a trait that is negatively correlated with dexterity [18, 19]. This may also explain some of the cross-national differences observed on PS tests. Another factor that may potentially affect processing speed is reading and writing skills, especially on tests that use digits and letters as stimuli. Studies such as the OECD PISA [20] based on very large samples (*n* > 100,000) find that females have better reading and writing skills than males. Lynn and Mikk [21] estimated the male/female gap to be *d* = 0.041 in the PISA 2010 study, and women are faster in handwriting in languages with very different orthographic systems, such as English [22], Chinese [23], and Japanese [24]. The female superiority has been explained by the fact that females study more and do more homework [21]. Increases in PS over generations [25] also suggests that practice effects based on rising educational levels may underlie observed differences in PS scores.

Differences in PS observed between nations and across gender suggest that there are several factors that affect PS and that these factors may act in different directions. For example, Finnish students have consistently ranked among the top five nations in reading and writing skills in the PISA assessments between 2005 and 2018, while Finnish standardization samples have consistently had significantly lower PS scores on the different versions of the WAIS as compared to U.S. and European standardization samples [13, 26]. It has also been found that males perform better than females on some types of speed tests, but females perform better on others. Males tend to be faster on fairly simple tasks such as reaction time tests and finger tapping, while females outperform males in rapid naming and in tests involving clerical-type skills [27–29]. In addition to being related to general intelligence, dexterity and reading and writing skills, speed tests are also affected by test-taking attitudes. According to Erdodi et al. [30], a test profile with a PSI score significantly lower than scores on the reasoning indexes may be caused by poor test-taker effort.

When different factors that may counteract each other are studied in separate studies using separate samples, it is difficult to analyze the effects of each factor. In the present study, the effects of a wide selection of factors on processing speed were analyzed in a sample of outpatients. The dependent variable was processing speed, measured by performance on the WAIS IV processing speed subtests (Digit Symbol Coding, Symbol Search), and the independent predictors were age, sex, height, weight, education, type of medication taken, gross manual dexterity, fine motor speed, grip strength, depression, performance on tests of nonverbal reasoning, verbal reasoning, working memory, and logical verbal memory, and 15 different personality traits.

The goal of the study was to explore the effects of psychological and physiological factors on processing speed in a study design that provides the opportunity to control for the major confounding factors of education and gender. It was hypothesized that sex differences in processing speed may be explained by factors that (a) have a positive correlation with PS when sex is controlled for, and (b) show a female advantage in population samples.

## Method

Subjects were 130 outpatients of the Medical Rehabilitation Clinic of Oulu University Hospital. The patients had been referred to the clinic for health assessment by doctors from health care centers, occupational health clinics, and from other clinics from the University hospital. The subjects had been diagnosed with one or several disorders that affected their ability to work. Most patients had been on a lengthy sick leave prior to the referral. At the MRC, the patients were assessed by a team composed of a medical doctor, a physiotherapist, an occupational therapist, and a psychologist. These assessments are paid for by the National Institute for Pensions or by private insurance companies. In most cases, the assessments result in recommendations for medical rehabilitation or treatment (physiotherapy, psychotherapy), occupational rehabilitation (changing occupation, studying for a physically or psychologically more suitable profession), or for a disability pension. All MRC patients from 2014 to 2018 that had taken the WAIS processing speed subtests and a test of fine motor speed or test of manual dexterity were included in the original sample (n ≈ 180). Patients with a significant neurological (epilepsy, MS-disease), or upper limb disorder were excluded from the study sample. Patients with a minor upper limb ailment that permitted pencil use were included in the sample. One criterion for exclusion was a very low (< -3sd) combined (dominant + nondominant hand) score on the Purdue Grooved Pegboard test (a test of fine motor speed) [31], or on the Box and Block test (a test of gross motor dexterity) [32]. Persons with a psychotic level psychiatric disorder or intellectual disability were also excluded from the study sample.

As shown in Table 1, patient diagnoses were classified into three groups: 113 patients in the sample had a diagnosis of musculoskeletal disease, 40 patients had a psychiatric diagnosis, and four had a neurological diagnosis. The medication patients were taking was categorized dichotomously: patients taking opiate medication (*n* = 48) and patients not taking opiate medication (*n* = 82). Patients’ educational background was also divided into two categories: patients that had completed high school (at least 12 years of education, *n* = 24) and patients with basic education (at least 9 years of education, *n* = 106). Differences in the proportion of males and females in diagnostic categories were small (effect size h < 0.40) and none reached statistical significance.

**Table 1:** Diagnoses, medication, and education

	Males (n = 49)	Females (n = 81)	Total (n = 130)	F(%) − M(%) difference		
				z	*p*	Effect size h
*Diagnosis*						
Psychiatric	15 (31%)	25 (31%)	40 (31%)	0.03	ns	0
Musculoskeletal	39 (80%)	74 (91%)	113 (87%)	1.92	0.054	0.32
Neurological	2 (4%)	2 (2%)	4 (3%)	0.52	ns	0.12
*Medication*						
Opiates	21 (43%)	27 (33%)	48 (37%)	1.09	ns	0.21
Psychiatric	11 (22%)	28 (35%)	39 (30%)	1.46	ns	0.29
*Education*						
Basic, 9–12 years	44 (90%)	62 (77%)	106 (82%)	1.89	0.059	0.36
> 12 years	5 (10%)	19 (23%)	24 (18%)	1.89	0.059	0.36

Table 2 shows the psychological and physical measurements that were included in the analysis.

**Table 2:** Psychological and physiological measurements, means

	Males (n = 49)			Females (n = 81)			M/F difference p	Effect size d	Population mean males	Population mean females	
	Mean	SD	n	Mean	SD	n					
Age	42	10.3	49	43	9.8	81	ns	0.1			
Height, cm	178	6.7	49	163	6.3	81	0.001	2.3	178	165	a
Weight, kg	93	19.6	49	80	20.7	81	0.001	0.65	87	73	a
BMI	29.4	5.7	49	29.7	7.6	81	ns	0.05	27.7	27.5	a
Beck Depression Index BDI II	14	10.8	33	15.6	9.7	66	ns	0.16	7	7	c
WAIS IV Processing speed PSI	86	14.3	43	97	16.6	75	0.001	0.71	96	103	d
WAIS IV Digit Symbol Coding	7.1	2.6	41	9.5	3.3	73	0.001	0.81	9	10.6	d
WAIS IV Symbol Search	8.0	3.0	42	9.4	3.1	74	0.023	0.46	9.4	10.3	d
WAIS IV Block Design	10.3	2.3	32	9.8	3.1	49	ns	0.18	9.8	10.1	d
WAIS IV Matrix reasoning	9.5	2.9	27	9.2	3.4	48	ns	0.1	10.1	9.9	d
WAIS IV Perceptual reasoning index PRI	100	14.0	41	98	16.8	69	ns	0.13	100	100	e
WAIS IV Similarities	9.4	2.2	37	9.3	3.2	65	ns	0.04	9.9	10.1	d
WAIS IV Digit Span	9.2	2.0	27	9.1	3.0	41	ns	0.04	9.9	10.1	d
WMS III Logical Memory	9.8	2.6	43	11.5	3.3	72	0.007	0.57	10	10	f
WMS III Logical Memory Delayed	9.8	2.5	42	11.1	3.3	70	0.03	0.44	10	10	f
Grooved Pegboard Dominant hand, seconds	76	19.8	27	74	21.1	57	ns	0.1	72	63	g
Grooved Pegboard Nd hand, s	82	20.8	26	80	23.9	55	ns	0.09	79	70	g
Grooved Pegboard (D + Nd), s	158	38.2	27	155	42.7	57	ns	0.07	151	133	g
Box and Block D hand, points	57	15.1	31	64	12.7	64	0.03	0.50	73	77	h
Box and Block Nd hand, points	57	14.4	30	63	13.3	60	0.08	0.43	71	74	h
Box and Block (D + Nd), points	115	29.5	31	126	25.0	61	0.09	0.40	144	151	h
Grip strength D Hand, kg	45	11.8	34	24	9.2	70	0.001	1.98	53	32	i
Grip strength Nd hand, kg	40	11.7	32	23	9.3	69	0.001	1.61	51	28	i
Grip Strength (D + Nd), kg	90	19.9	34	48	16.6	70	0.001	2.3	104	60	i
One foot stand, s	65	46.1	43	62	45.8	70	ns	0.06	80.2	80.4	j
PRF Dominance	7	4.6	34	5.2	4.2	63	0.06	0.41	11.3	9.8	k
PRF Exhibition	6.1	4.4	34	5.0	3.6	63	ns	0.23	9.8	9.6	k
PRF Achievement	9.3	3.5	34	7.8	3.1	63	0.03	0.45	10.6	10.4	k
PRF Succorance	7.9	2.9	34	9.5	3.3	63	0.02	0.51	10.2	11.0	k
PRF Affiliation	7.6	4.7	34	8.3	3.8	63	ns	0.16	12.5	12.7	k
PRF Nurturance	8.5	3.7	34	11.1	2.8	63	0.001	0.80	8.8	10.3	k
PRF Cognitive structure	9	2.8	34	8.8	2.7	63	ns	0.07	8.6	8.4	k
PRF Order	8.1	4.5	34	10.7	3.8	63	0.003	0.62	8.7	9.4	k
PRF Impulsivity	6.1	3.3	34	7.8	3.8	63	0.02	0.48	5.6	7.3	k
PRF Defendence	4.7	2.5	34	5.5	2.0	63	ns	0.35	5.7	6.7	k
PRF Anxiety	6.8	3.6	34	7.8	3.0	63	ns	0.30	3.8	5.9	k
PRF Guilt	7.8	5.6	34	10.1	5.2	63	0.04	0.43	6.0	6.6	k
PRF Aggression	7.3	3.0	34	7.4	3.8	63	ns	0.03	7.7	8.5	k
PRF Harm avoidance	8.4	3.8	34	11.3	4.3	63	0.001	0.71	6.3	8.8	k
PRF Sentience	7.1	2.9	34	9.6	4.3	63	0.001	0.68	7.9	10.4	k
PRF Desirability	7.1	3.0	34	7.8	2.4	63	ns	0.26	6.3	6.3	k

Reference a = [39], b = [40], c = [37], d = estimate based on Wechsler [41] standardization data, e = estimated (Block + Matrix) PRI, f = [36], g = [31], h = [42] i = [33], j = [34], k = [38]

WAIS IV and WMS scaled subtest scores, WAIS IV index scores, PRF raw scores

ns = *p* > 0.1

Patient age, sex, height, weight and Body-Mass index (BMI) were registered. Test results from the occupational therapy assessment included scores on: (1) the Purdue Grooved Pegboard (left and right hand), a test of fine motor speed, and (2) the Box and Block test (left and right hand), a test of gross motor dexterity. The physiotherapy tests included: (1) grip strength, measured with a Jamar dynamometer [33] and (2) one foot stand (right and left foot) [34].

The following psychological tests were included in the analysis: (1) the WAIS IV [35] Block Design and Matrix reasoning subtests that measure nonverbal reasoning, the Vocabulary and Similarities subtests that measure verbal comprehension, and the Digit Symbol Coding and Symbol Search subtests that measure processing speed; (2) the Wechsler Memory Scale III [36] Logical Memory I and II subtest that measures immediate and delayed verbal memory, and Digit Span subtest that measures working memory; (3) the Beck Depression Inventory II [37]; and (4) The Personality Research Form [38], a popular personality test.

Information for all 130 patients was available only for age, sex, height, weight, body-mass index, diagnosis, and medication. Table 2 shows the number of male and female patients that had taken each test.

Microsoft Excel 2010 was employed to determine the descriptive statistics (means, standard deviations) of the data. Further statistical analyses were performed using IBM SPSS Statistics version 25. Differences in mean scores between males and females were analyzed by t-tests. Correlations (Pearson) between the variables were determined and further correlational analysis controlling for sex, education and the use of opiate painkillers was performed. Multiple stepwise linear regression analysis was performed to analyze the contributions of the psychological, physiological, and background factors to PS.

The study design was approved by the ethical committee of the Northern Ostrobothnia Hospital District (permit number 111/2019).

## Results

Table 2 shows means for the cognitive and physiological tests and measurements. As Table 2 indicates, the subjects were of average height but many subjects were overweight and the mean BMI mean was close to the criterion for obesity (> 30). Nonverbal and verbal reasoning scores were close to the national mean, as were memory test scores. Psychomotor processing speed was slightly below the national mean in men. Gross manual dexterity (Box and Block) was low in both men and women, and fine motor speed (Grooved Pegboard) was lower than the population mean for women. The mean Beck Depression Inventory score for both males and females reached the limit (14 points) for mild depression. The personality test scores indicate that the subjects were more introverted, less ambitious, less social, more anxious and more risk avoidant as compared to the PRF standardization sample of Finnish college students.

Statistical significance of the differences between the study sample and the general population was not calculated because of the incomplete standard deviation data provided by test manuals. Significant male–female differences were found in processing speed, logical verbal memory, grip strength, gross manual dexterity (dominant hand), and on several personality scales. As Table 2 shows, these differences roughly conform to sex differences observed in test standardization samples. One major exception is that there was not a statistically significant sex difference in fine motor speed (Pegboard test) in the study sample, as compared to the fairly large male/female gap in the general population. In the study sample, males were slightly slower on the Pegboard test, while females are remarkably faster in the general population. The male/female difference in the study sample is not much larger if medians rather than means are compared, 70/67 s for male/female dominant hands, 77/75 s for nondominant hands and 149 s/141 s for both hands combined.

As Table 3 shows, processing speed has a moderate positive correlation with perceptual reasoning, verbal comprehension, verbal memory, and fine motor speed. Weak but statistically significant correlations were found between PS and gross manual dexterity and working memory. Only a few personality traits were found to have a statistically significant correlation with PS: dominance, exhibition, defendence, and sentience. Weak to moderate correlations between PS and education, were found. With very few exceptions, correlations involving Digit Symbol Coding were higher than those involving Symbol Search.

**Table 3:** Correlation between processing speed and selected psychological and physiological measurements with and without controlling for sex, education, and medication

	Digit Symbol Coding	Symbol Search	Processing Speed Index	Controlling for sex, education, and medication		
	DS	SS	PSI	DS	SS	PSI
Age	− 0.03	− 0.06	− 0.04	− 0.09	− 0.08	− 0.09
Height	− 0.16	0.03	− 0.07	0.16	0.29**	0.25**
Weight	− 0.20*	0.01	− 0.08	− 0.07	0.08	0.02
Body-mass index	− 0.15	− 0.01	− 0.09	− 0.18	− 0.03	− 0.09
Education	0.26*	0.12	0.21*			
Medication	0.15	0.02	0.09			
Beck Depression Index	− 0.09	− 0.03	− 0.04	− 0.07	− 0.03	− 0.05
WAIS Block Design	0.50***	0.30**	0.50***	0.54***	0.48***	0.54***
WAIS Matrix reasoning	0.35**	0.21	0.37**	0.37**	0.29*	0.38**
WAIS Perceptual reasoning index	0.47***	0.36***	0.47***	0.49***	0.38***	0.48***
WAIS Similarities	0.35***	0.19	0.28**	0.32**	0.17	0.25*
WMS Digit Span	0.30*	0.28*	0.30*	0.37***	0.30***	0.36***
WMS Logical memory immediate	0.46***	0.34***	0.43***	0.43***	0.35***	0.42***
WMS Logical memory delayed	0.49***	0.38***	0.47***	0.28*	0.27*	0.28*
Box and Block Dominant hand	0.25*	0.12	0.20	0.21	0.10	0.16
Box and Block Nondominant hand	0.33**	0.21	0.31**	0.31**	0.22	0.28*
Box and Block combined	0.28*	0.16	0.24*	0.26*	0.15	0.22
Grooved Pegboard DH	− 0.43***	− 0.20	− 0.39***	− 0.42***	− 0.34**	− 0.39**
Grooved Pegboard NDH	− 0.47***	− 0.30*	− 0.46***	− 0.47***	− 0.40**	− 0.46***
Grooved Pegboard combined	− 0.47***	− 0.39***	− 0.45***	− 0.47***	− 0.39**	− 0.45***
Grip strength DH	− 0.09	0.02	− 0.09	0.20	0.12	0.15
Grip strength NDH	− 0.10	− 0.03	− 0.09	0.14	0.13	0.13
Grip strength combined	− 0.13	− 0.10	− 0.14	0.18	0.08	0.13
One foot stand	0.16	0.13	0.15	0.14	0.14	0.14
PRF Dominance	0.30**	0.18	0.25*	0.39***	0.27*	0.33*
PRF Exhibition	0.24*	0.06	0.15	0.31*	0.09	0.21
PRF Achievement	0.01	− 0.06	− 0.02	0.10	− 0.01	0.05
PRF Succorance	0.07	0.06	0.06	− 0.06	0.00	− 0.04
PRF Affiliation	0.09	− 0.04	0.02	0.06	− 0.06	− 0.01
PRF Nurturance	0.15	0.10	0.13	0.04	0.03	0.04
PRF Cognitive structure	− 0.16	− 0.04	− 0.10	− 0.19	− 0.05	− 0.12
PRF Order	0.13	0.16	0.15	0.06	0.11	0.09
PRF Impulsivity	0.10	0.03	0.07	0.06	− 0.01	0.02
PRF Defendence	0.19	0.22*	0.22*	0.17	0.20	0.20
PRF Anxiety	0.03	0.09	0.07	− 0.01	0.06	0.03
PRF Guilt	0.03	0.01	0.02	− 0.01	− 0.06	− 0.03
PRF Aggression	0.10	0.12	0.12	0.11	0.12	0.12
PRF Harm avoidance	− 0.07	0.01	− 0.03	− 0.22*	− 0.06	− 0.15
PRF Sentience	0.30**	0.12	0.23*	0.18	0.04	0.11
PRF Desirability	− 0.11	− 0.08	− 0.11	− 0.15	− 0.10	–0.14

***p < .001; **p < .01; *p < .05

When controlling for sex, education, and use of opiate painkillers, correlations between PS and reasoning, memory, and fine motor speed remained moderately high, and those between extraversion (dominance, exhibition) and PS, and between gross manual dexterity and PS, had weak but statistically significant correlations. Contrary to expectations, height was found to have a positive correlation with processing speed.

Twelve variables (sex, and variables with > 0.30 correlation with the Coding subtest) were included in further analysis. Three models emerged in stepwise regression analysis: a model with one predictor (Block Design), a two variable model (Block Design; Sex), and a three variable model (adjusted R^2^ = 0.515) with Block design (β = 0.51, t = 4.61, *p* = 0.000), Sex (β = 0.39, t = 3.57, *p* = 0.001) and fine motor speed (Purdue pegboard, dominant hand) (β =  − 0.37, t =  − 3.39, *p* = 0.002) as three independent predictors of processing speed.

## Discussion

Theoretically, a factor that explains the male/female gap in processing speed should (a) have a positive correlation with PS when sex is controlled for, and (b) show a female advantage in population samples. Among the factors analyzed in the present study, only fine motor speed meets this criterion, as females are faster on the Pegboard test in population samples and the test score has a moderate correlation with WAIS PS scores. Obviously, this observation is not very helpful and simply pushes the need for explanation just one notch further: why should females have higher fine motor speed as compared to males? Our data suggests that female superiority in PS and fine motor speed is not associated with female advantages in nonverbal or verbal reasoning or working memory. In our sample, females did have higher logical memory scores than men; however, in the Finnish standardization sample, there was no sex difference. Height and extraversion (dominance, exhibition) correlated with PS when sex was controlled for; however, in the sample as well as in the general population men are taller and more extroverted than are women. The positive correlation between height and PS is especially intriguing, as previous studies suggest that small hand size is correlated with faster PS (Symbol Search) [17] and tall people tend to have larger hands. In the study sample, no significant correlation was observed between height and fine motor speed (*r* = 0.009).

The regression analysis resulted in a very simple model: among the multitude of predictors studied only nonverbal reasoning, fine motor speed, and sex were independently associated with PS. For example, the memory tests had high positive correlations with PS, however, it seems that the Block Design score is a better measure of the underlying factor that is associated with PS. This factor, most likely, is general intelligence [47].

In conclusion, none of the factors analyzed in the present study seem to explain the male/female gap in PS in an adequate way. It may be that the factors underlying gender differences in PS are not psychological but neurological or physiological in nature and a wider variety of measures from these disciplines are needed in order to explain the gender gap.

In WAIS standardization samples, the male/female gap has consistently been roughly 0.3 sd’s, or 5 IQ points across nations and across test versions [4, 10–12]. There is abundant evidence from other neuropsychological tests that females outperform males in fine motor speed [29]. While 5 IQ points is, in the clinical setting, a fairly insignificant difference, combined with the standard error of measurement and other factors that affect the reliability of psychological testing, this inaccuracy may contribute to invalid conclusions. Arguably, test norms for psychomotor processing speed tests should be separate for males and females.

The results concerning personality traits do not explain the male/female gap; rather, they may even make it more difficult to explain, as extraversion and risk taking were shown to be positively correlated with PS and males are slightly more extroverted and less risk avoidant on the PRF scales than are females. However, this observation may be relevant for explaining cross-national differences in PS discussed above [13]**.** Nations such as the Scandinavian countries where introverted and cautious behavior is the cultural norm (reflected, for example, in traffic deaths and law enforcement [43, 44]) may show a lower Perceptual Reasoning/Processing Speed ratio in standardization samples as compared to nations where more extroverted and courageous behavior is viewed positively (such as the U.S.).

The major limitation of the present study is the nature of the sample. In general, the sample was quite small and had an uneven number of males and females. All participants had a physical or psychological condition that affected their ability to work. While persons with major neurological, psychiatric or upper limb disorders were excluded from the sample, 48 patients in the sample used opiate medication for musculoskeletal disorders. Pain medication that affects the central nervous system as well as pain itself may have affected the test performance of the patients [45]. Likewise, psychiatric medication, that was used by 39 participants may have affected the test results [46]. The educational level of the patients was also lower than that of the general population, and many had been employed in manual labor occupations.

Another limitation of the study is that processing speed was measured using one test instrument only, the Wechsler test. We cannot rule out the possibility that some of the results may be test-specific to some extent. These features of the sample and methods call for caution when judging the generalizability of the results. However, the fact that the magnitude of the male/female gap was roughly the same in the study sample as in the general population suggests that the results have external validity.

Since our observations remain inconclusive, we suggest that in future studies a wider variety of psychological, neurological, and physiological tests and measures should be used. Furthermore, in the ideal case, the study sample should be composed of healthy persons. Unfortunately, convenience samples or clinical samples that meet these criteria most likely do not exist. However, the results of the present study do seem to narrow the pool of psychological variables that need be further studied. For example, it seems that personality traits have little independent effect on processing speed.

## Conclusions

The finding that processing speed had a moderate positive correlation with perceptual and verbal reasoning, memory, and fine motor speed, is consistent with prevailing theories of intelligence. In the present study, sex and education were also found to be related to processing speed, in line with earlier research. When controlling for sex and education, the personality trait of extraversion had a weak positive correlation with processing speed, while no significant associations were found between other personality traits and processing speed. Female superiority in psychomotor processing speed is associated with female superiority in fine motor speed; however, the underlying cause of the male/female gap in these skills remains unknown. One interpretation of these results is that the factors underlying sex differences in processing speed are not psychological but neurological or physiological in nature, and therefore a wider variety of measures from these disciplines may be needed to explain the gender gap. As the male/female difference in processing speed has been solidly documented in previous research and cannot be exhaustively explained by other psychosocial factors, test norms for psychomotor processing speed tests should, arguably, be separate for males and females.

## Data Availability

The data utilized to support the findings of this study are available from the corresponding author upon reasonable request.

## Abbreviations

BDI: Beck Depression Inventory
BMI: Body-Mass Index
PISA: Programme for International Students Assessment
PS: Processing Speed
PRF: Personality Research Form
PRI: Perceptual Reasoning Index
VCI: Verbal Comprehension Index
WAIS: Wechsler Adult Intelligence Scale
WMI: Working Memory Index
